# Consequences of a Government-Controlled Agricultural Price Increase on Fishing and the Coral Reef Ecosystem in the Republic of Kiribati

**DOI:** 10.1371/journal.pone.0096817

**Published:** 2014-05-12

**Authors:** Sheila M. W. Reddy, Theodore Groves, Sriniketh Nagavarapu

**Affiliations:** 1 Central Science Division, The Nature Conservancy, Arlington, Virginia, United States of America; 2 Environmental Change Initiative, Brown University, Providence, Rhode Island, United States of America; 3 Department of Economics, University of California-San Diego, La Jolla, California, United States of America; 4 Department of Economics and Center for Environmental Studies, Brown University, Providence, Rhode Island, United States of America; Dauphin Island Sea Lab, United States of America

## Abstract

**Background:**

Economic development policies may have important economic and ecological consequences beyond the sector they target. Understanding these consequences is important to improving these policies and finding opportunities to align economic development with natural resource conservation. These issues are of particular interest to governments and non-governmental organizations that have new mandates to pursue multiple benefits. In this case study, we examined the direct and indirect economic and ecological effects of an increase in the government-controlled price for the primary agricultural product in the Republic of Kiribati, Central Pacific.

**Methods/Principal Findings:**

We conducted household surveys and underwater visual surveys of the coral reef to examine how the government increase in the price of copra directly affected copra labor and indirectly affected fishing and the coral reef ecosystem. The islands of Kiribati are coral reef atolls and the majority of households participate in copra agriculture and fishing on the coral reefs. Our household survey data suggest that the 30% increase in the price of copra resulted in a 32% increase in copra labor and a 38% increase in fishing labor. Households with the largest amount of land in coconut production increased copra labor the most and households with the smallest amount of land in coconut production increased fishing the most. Our ecological data suggests that increased fishing labor may result in a 20% decrease in fish stocks and 4% decrease in coral reef-builders.

**Conclusions/Significance:**

We provide empirical evidence to suggest that the government increase in the copra price in Kiribati had unexpected and indirect economic and ecological consequences. In this case, the economic development policy was not in alignment with conservation. These results emphasize the importance of accounting for differences in household capital and taking a systems approach to policy design and evaluation, as advocated by sustainable livelihood and ecosystem-based management frameworks.

## Introduction

Governmental and non-governmental organizations are increasingly interested in understanding the connections between economic development and environmental sustainability. For example, the Millenium Development Goals [1] and the Millenium Ecosystem Assessment [2] suggest that economic development and environmental sustainability goals are inextricably linked because people both depend on and impact the environment and natural resources. However, our understanding of these linkages are based on fairly limited empirical evidence due to the institutional, disciplinary, and logistical challenges associated with taking a systems approach to policy and program design and evaluation [3], [4]. As a result of this incomplete understanding, policies and programs may not effectively advance the economic and environmental goals of managers [5].

Economic development policies may have unexpected economic and ecological consequences within and beyond the sectors they were meant to target. Households in developing countries often participate in multiple livelihood activities in order to enhance their ability to maintain income or consumption levels despite changing economic and environmental conditions [5], [6]. This means that economic development policies focused on a single sector are likely to affect activities in another sector [7]. The effect of these policies may also extend throughout ecosystems because households in developing countries are often dependent on harvesting natural resources [8], which can affect non-target resources through ecological interactions [9]. However, the ecological effects of economic development policies often go unnoticed because they are rarely evaluated, even in the context of integrated conservation-development policies [10]. Moreover, these economic and ecological consquences are likely to vary across households since housholds are not homogeneous: Previous studies show that the magnitude and direction of a household's response to economic development policies may be affected by their levels of and access to capital, education levels, subsistence requirements, or preferences [11]–[16]. The sustainable livelihood approach and ecosystem-based management provide practical frameworks to improve rural policy development by enabling policy-makers to systematically consider the conditions created by these different factors [5], [17]. Yet, managers still have few empirical examples to look to that document both the economic *and* ecological outcomes of economic development policies.

This paper helps fill this knowledge gap with a case study of the effect of a government increase in the price of the primary agricultural product (copra) on agriculture, fishing, and the coral reef ecosystem in the small Pacific island nation of the Republic of Kiribati. Like many fishing households worldwide [5], Kiribati households participate in both agriculture and fishing and regularly shift labor between them in response to changing conditions [6]. Given this livelihood strategy, we expected that the copra price increase would not only impact copra but that it would also impact fishing through changes in labor allocation, with variable responses across households. We also expected that resulting changes in fishing labor would affect fish stocks and the algae that fish eat as well as the algae's competitors, corals [18], [19].

Standard economic theory, as well as a livelihood framework, provides some basic predictions for the direction of the effects of the copra price increase on copra labor, fishing labor, and the coral reef ecosystem. For instance, we expect that the copra price increase has two main effects: first, it increases the marginal revenue product of copra labor (equivalent to a wage and defined as the copra price x marginal quantity of copra produced) at any given level of copra labor; second, it increases household income. The former effect increases the shadow price (i.e., the implicit cost) of leisure and the shadow price of fishing labor. This encourages households to increase labor in copra and reduce leisure and time spent fishing. The latter effect on household income will increase consumption of all normal goods and leisure, which could in turn reduce copra labor and fishing labor. Both effects lead to the prediction that fishing will decline, which would then relieve pressure on the coral reef ecosystem.

In settings like Kiribati, however, this standard reasoning may not be sufficient. Kiribati is a mixed subsistence-cash economy with restricted markets for credit, labor, and goods; relatively healthy coral reef fisheries; and a culture that derives important non-monetary benefits from fishing, like many other fishing cultures [15]. Since credit markets are limited, the increase in income could relieve credit constraints and allow households to invest in new fishing equipment or land when they could not before [20]. However, the markets for fishing equipment and, especially, land are limited so household responses may be constrained by their current capital assets [21]. Since labor cannot be hired from outside, additional labor in copra should result in concomitant decreases in labor in fishing or leisure [21], unless this is counteracted by an income effect. Since the market for fish is limited, the increased demand for fish that comes with greater income must be primarily met by increasing local production [22], [23], which could draw labor back into fishing. Importantly, the relatively good condition of the fishery suggests that fishermen's responses to the copra price increase are not likely to be strongly enhanced by declining fish stocks [5]. The importance of non-monetary benefits from fishing in Kiribati suggests that increased household income, and hence increased leisure, may actually mean increased fishing. Considering these possibilities, the effects of the copra price increase on fishing labor—and therefore the coral reef ecosystem– are theoretically ambiguous and may vary in important ways across households with differing capital assets and preferences. Therefore, the consequences of the copra price increase must be examined empirically.

To examine these possibilities, we estimated the effect of the copra price increase on labor decisions using household survey data and linked the effects of these labor decisions to changes in interacting ecological stocks using ecological survey data. In our analysis, we used household land to identify differences in the responses of households to the price increase. The paper is organized as follows. First, we present background on the case study. Second, we present the methods and results from the empirical evaluation of economic and ecological outcomes. Third, we finish with a discussion of the results and conclusions.

## Background

### The Agricultural Price Increase

**Figure 1:**
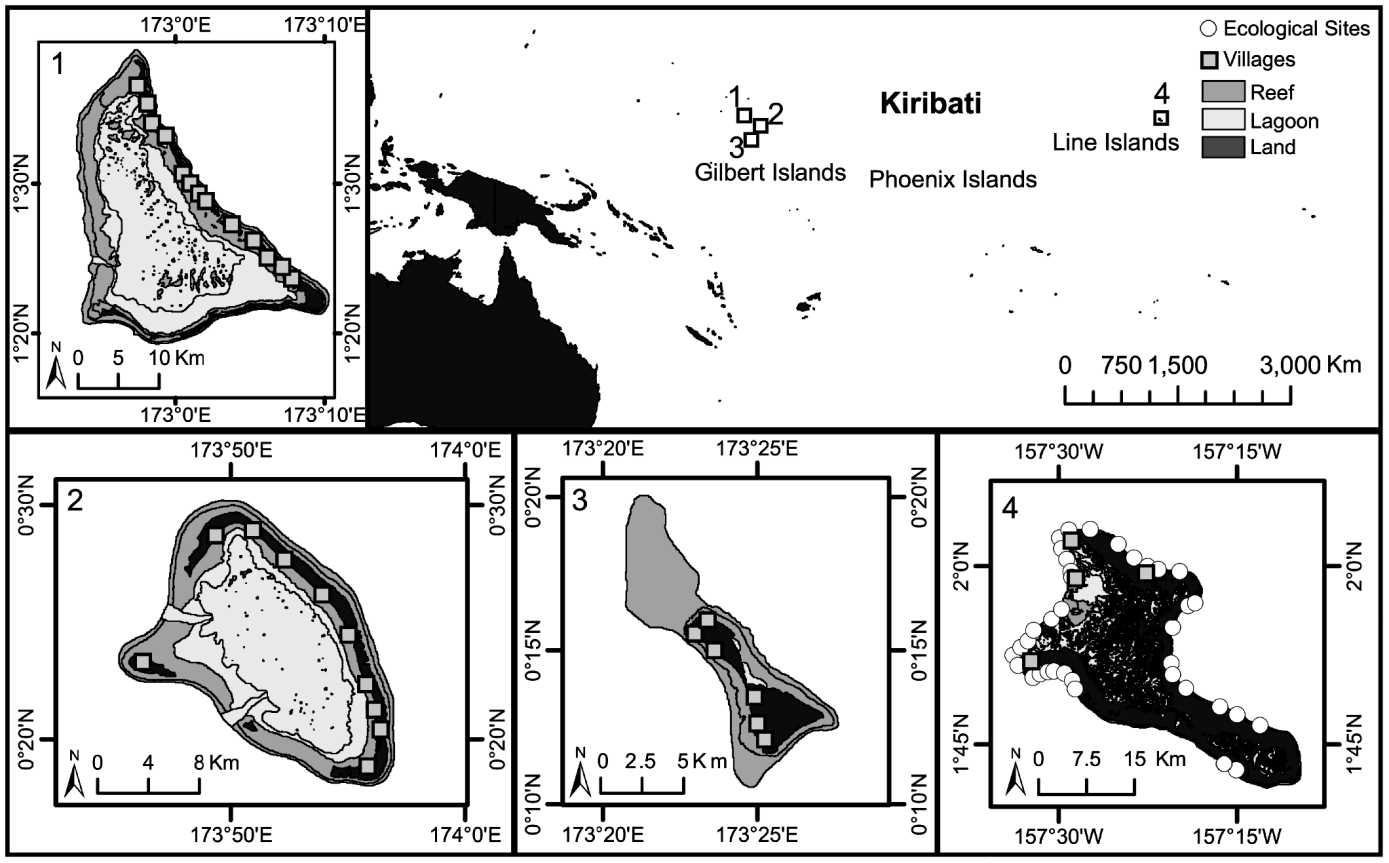
Map of the Republic of Kiribati showing study islands with villages and ecological sites.

Copra is the dried meat of coconuts and it is used to produce coconut oil. In Kiribati, households sell copra to the government, which in turn exports the crop. In 2003 and 2004, the government increased its buying price for copra as part of a social welfare program implemented by the Ministry of Finance. The large increases in the copra price in 2003 and 2004 followed a presidential election where promises for price increases were made during the campaign. The Ministry of Fisheries supported the program because it expected that the increases in the copra price would help relieve pressure on the coral reefs by increasing labor in copra and decreasing fishing, as the standard economic reasoning noted above would predict [24]. This program resulted in a 9% increase in the copra price in the main Gilbert Islands and a 17% increase in the Line Islands (Fig. 1, Table 1). The spatial variation in the price increase is due to the fact that the copra price in the Line Islands had been historically lower than in the Gilbert Islands because of their greater distance from the capital, Tarawa. In 2004, the copra price was increased by another 21% in all islands (Table 1).

**Table 1:** The government-controlled copra buying price (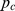) and the market fish price (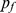) (2001 AUD/kg)^1^.

Year	Gilbert Islands				Lines Islands			
	*p_c_*	% Δ	*p_f_*	% Δ	*p_c_*	% Δ	*p_f_*	% Δ
2001	0.45	NA	0.77	NA	0.42	NA	0.66	NA
2002	0.43	−5	0.74	−4	0.40	−5	0.62	−6
2003	0.47	9	0.75	1	0.47	17	0.61	−2
2004	0.57	21	0.78	4	0.57	21	0.56	−8
2005	0.57	0	0.82	5	0.57	0	0.58	4
2006	0.58	2	0.86	5	0.58	2	0.61	5

1 The government-controlled copra price is the same across households and the fish price is an average reported across households. Note that a regression of the natural log of fish price on the natural log of copra price that includes household and island fixed effects and clustered standard errors by household-village-year shows a small, positive relationship (0.14, *SE* = 0.05, p<0.001). A Spearman rank correlation yields the same results (Table S1).

Three features of the copra price increase in Kiribati lend itself to empirical evaluation. First, the spatial and temporal variation in the copra price, as well as cross-household variation in land, enabled us to identify changes in labor in fishing and copra that may have resulted from the copra price change as well as heterogeneous changes across the distribution of household land in coconut production. Note that no significant temporal variation in household land was found over the study period, which reflects the limited land market. Second, the policy had limited potential for selection bias because the price increase was unexpected, it did not target specific areas based on fish stocks, and the majority of households engage in or have access to fishing and copra. Third, an extreme spatial gradient in fishing pressure on the largest island, Kiritimati, enabled us to translate estimated changes in fishing labor into changes in the coral reef ecosystem (Fig. 1). This approach is both necessary and preferable to collecting real-time ecological data because real-time data was not available and fishing is known to have lagged effects on coral reef ecosystems [25].

### The Fishing-Agricultural Household

The Republic of Kiribati is a small island nation in the Central Pacific (Fig. 1). Our household survey results show that the majority of households (58%) participate in fishing and copra production and do not get income from any other activity. In a given year, we found that some households may fish and not produce copra (32%) but it is uncommon for households to produce copra but not to fish (5%). The average household is made up of 6–7 people (2 adult males) and spends a similar amount of time fishing (20 person*hrs/wk) as they do working in copra (19 person*hrs/wk) (Table 2, see also Table S1). Although men do the majority of the fishing, we observed that men and women as well as children participate in copra and some types of fishing. Moreover, these activities often are scheduled at different times (e.g., copra production during the day with fishing at night 2–3 times per week). In a year, a household may earn on average $2,305 (2001 AUD) per year from copra and $1,653 (2001 AUD) per year from fishing (Table 2). Households in the top 50^th^ percentile for land in coconut production get the majority of their income from copra, while households in the bottom 50^th^ percentile for land in coconut production get the majority of their income from fishing (Table 2).

**Table 2:** Descriptive statistics on household survey data and environmental data from four islands in Kiribati over the period 2001–2006^1^.

Variable	[Units]	All Households					Households with Land ≤50th Percentile (3 acres)					Households with Land >50th Percentile (3 acres)				
		Mean	N	SD	Max	Min	Mean	N	SD	Max	Min	Mean	N	SD	Max	Min
*p_c_*	[2001 AUD/kg]	0.51	1627	0.07	0.58	0.4	0.51	852	0.07	0.58	0.4	0.51	775	0.06	0.58	0.4
*p_f_*	[2001 AUD/kg]	0.73	1627	0.13	0.95	0.51	0.71	852	0.14	0.95	0.51	0.75	775	0.12	0.95	0.51
*L_f_*	[persons*hrs/yr]	0.57	1627	1.15	10	0	0.55	852	1.28	7.5	0	0.6	775	0.98	10	0
*L_c_*	[persons*hrs/yr]	0.51	1574	0.63	7.88	0	0.59	833	0.75	7.88	0	0.41	741	0.45	4	0
*L_other_*	[persons*hrs/yr]	0.2	1620	0.56	4.5	0	0.27	846	0.68	4.5	0	0.11	774	0.38	3	0
*I_c_*	[2001 AUD/yr]	2305.05	1620	4164.88	45625	0	1667.84	852	4889.28	45625	0	3011.96	768	3023.29	17784	0
*I_f_*	[2001 AUD/yr]	1653.34	1498	4102.45	62400	0	1953.61	778	4762.43	62400	0	1328.88	720	3213.69	29250	0
*I_other_*	[2001 AUD/yr]	782.67	1615	2385.05	37770	0	1124.63	852	2905.52	37770	0	400.81	763	1530.99	20800	0
HH that sell fish	[count]	0.48	1498	0.5	1	0	0.51	778	0.5	1	0	0.44	720	0.5	1	0
Spending on rice	[2001 AUD/yr]	708.77	1574	688.48	9504	0	797.45	828	568.1	3900	0	610.33	746	790	9504	0
Spending on fish	[2001 AUD/yr]	363.15	1584	669.6	5720	0	398.22	821	699.11	5720	0	325.42	763	634.62	5000	0
Coconut Land	[acres]	5.15	1627	6.35	37	0.01	1.24	852	1.01	3	0.005	9.44	775	6.96	37	3.25
HH Size	[count]	6.61	1627	3.15	19	1	7.17	852	3.57	19	2	5.99	775	2.47	15	1
Males	[count (15–60 yr)]	2.01	1627	1.34	8	0	2.28	852	1.5	8	0	1.71	775	1.07	7	0
Education	[yrs >primary]	1.96	1627	1.81	10	0	1.94	852	1.8	10	0	1.98	775	1.81	10	0
Rain	[mm/yr]	1733.99	1627	688.69	2998.81	547.26	1519.92	852	737.25	2998.81	547.26	1969.32	775	541.2	2998.81	547.26
Rain*_(t-1+t-2)_*	[mm/yr]	3146.29	1627	1451.69	5499.55	616.14	2686.37	852	1530.03	5499.55	616.14	3651.9	775	1168.11	5499.55	616.14
Reef Area	[km^2^]	372.29	1627	152.54	560.6	52.12	452.39	852	130.94	560.6	52.12	284.23	775	123.31	560.6	52.12
House	[1 = concrete]	0.14	1595	0.35	1	0	0.24	832	0.43	1	0	0.04	763	0.18	1	0
Boats	[count]	0.47	1627	0.84	6	0	0.49	852	0.94	6	0	0.44	775	0.71	3	0

1 The copra price is constant across households on the same island and is published by the government (see Table 1) Abbreviations: prices (*p*), labor (*L*), income (*I*), household (HH). Subscripts indicate copra (*c*), fishing (*f*), or other (*other*) labor or income. See Table S1 for Spearman rank correlations between the copra price and the fish price and these variables.

The local fishery is open access and fishing is primarily done on nearby coral reefs with simple technology, such as handlines, gillnets and canoes (sometimes with small motors) (Table 2). Fish are either consumed by the household or sold in local markets. Note that less than half (48%) of households sell fish in a given year (Table 2). Coconuts are harvested from trees on household land (Table 2). Little to no improvement is done to the land. Although copra plantations existed in Kiribati when it was a British Colony, copra production since independence in 1979 is done on household land and rarely involves fertilizer, clearing of undergrowth, or intentional planting of coconut trees. Coconuts are left to grow into trees or harvested opportunistically by household members. The meat is removed, often using implements fashioned from scrap metal, and dried on mats or racks made from coconut fronds or local wood. Copra is sold to local agents from the government-owned copra exporting companies. Cash from copra is primarily used to purchase rice, which is imported, to purchase a limited number of other food items (such as fish), to pay school or church fees, or to buy a limited number of durable goods. Average annual household spending on rice is $709 (2001 AUD) and spending on fish is $363 (2001 AUD).

Household labor allocation can respond quickly to changes in fishing or copra prices because most households have the requisite skills and capital for both activities. Almost all households own land with coconut trees and they also own fishing gear or share it with family members. Moreover, shifting labor allocation across fishing and copra is the primary way that households change their livelihood strategies because markets for land, labor, and credit are limited and there are few educational and occupational opportunities.

### The Coral Reef Ecosystem

The islands of the Republic of Kiribati are atolls that were formed by the growth of corals. Live corals in the lagoon and ocean waters surrounding these islands actively grow and build reef structures that protect people from storms, support an active subsistence fishery, and provide habitat for many interacting species [26]. Although corals support fisheries, fish also play a role in supporting healthy corals by eating corals' competitor, algae. As a result, overfishing has been a major cause of the shift from coral dominated to algal dominated reefs worldwide, together with nutrient pollution and other human impacts [18], [27]–[29]. While Kiribati has some of the world's most pristine reefs, other reefs in Kiribati have been transformed by fishing but are still relatively healthy compared to most coral reefs [18], [30]–[32].

## Methods

### Ethics Statement

This research was permitted by the Ministry of Environment, Lands and Agricultural Development, Government of the Republic of Kiribati, located at Bikenibeu, Tarawa, Kiribati.

The University of California- San Diego Institutional Review Board, part of the Human Research Protections Program, approved the household survey methods and consent form for this research (project record #060412). Using the approved form, we obtained informed consent from households prior to implementing the survey.

### Household Data and Analysis

In May and June 2007, we collected retrospective data (2001–2006) from heads of 277 households on four islands that were selected using a cluster sampling design, resulting in a 2% sample of the total number of households in the country (Fig. 1). The survey instrument was developed with input from officers from the Ministry of Finance and Ministry of Fisheries and pre-tested on 85 households on two islands in December 2006. The survey gathered information on household labor, income, education, capital ownership, and demographics (Table 2). In the cluster sampling design, each island was considered a different cluster. Islands were chosen based on the probability that a household would be drawn from a random sample of the population (households per island/total households). The target number of households surveyed at each island was proportional to the population of the island. Households from each village in a given island were surveyed in order to capture the variability within each island. Households are generally arranged linearly along the single or main road that runs the length of each island. This arrangement was used to randomly select households. A random number from 1–5 was chosen prior to entering a village and every n^th^ household was visited.

In addition to using household survey data, we used publicly available data on annual rainfall for the one degree cell associated with each island and coral reef area to help control for changes in the marginal productivity of labor across years and islands [33] (Table 2). The total coral reef area associated with each island was calculated as the sum of the lagoon and reef area queried from the Millennium Coral Reef Mapping Project validated maps provided by the Institute for Marine Remote Sensing, University of South Florida and Institut de Recherche pour le Développement, Centre de Nouméa, with support from NASA.

We expected the copra price increase to have a significant effect on labor allocation and ultimately resource stocks because households can readily shift labor across copra and fishing and the copra price increase represented a substantial increase in income. Using reported income from 2001 and assuming no change in labor, we calculated that the copra price increase could have resulted in a 6.2% increase ($580/year (2001 AUD)) in total annual income for households with land holdings in the bottom quartile and a 15.2% increase ($766/year (2001 AUD)) for households with land holdings in the top quartile.

To test the effect of the copra price change, we considered two linear models of copra 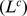 and fishing 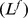
labor:
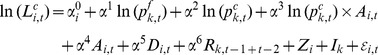


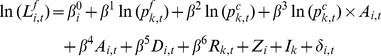
where labor allocation is predicted by island- (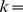
1 to 4) and year- (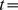
2001 to 2006) specific prices for copra (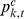) and fish (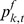), adjusted for inflation (Table 1), as well as the interaction between household land under cultivation for coconuts (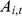) and the copra price. The models also include other variables that may affect the opportunity cost of time allocations: household land under cultivation for coconuts (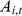), basic household demographic and socioeconomic variables (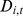), island-specific rainfall in the current year (for the fishing labor model) and summed over the two previous years (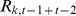) (for the copra labor model). For instance, households with more adult males and higher education levels may have a greater opportunity cost for copra labor. In contrast, when previous rainfall was high and households have lots of land, the opportunity cost of labor in fishing may be high. Note that we assume a two year lag between rainfall events and copra production because of the maturation time of coconuts [36], [37]. In contrast, we assume no lags between rainfall and fishing because current weather conditions are more important in determining whether people go out to fish. To control for fixed unobservable heterogeneity across households and islands, we included household fixed effects, 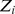, and island fixed effects, 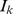. The island fixed effects were included because some households did change islands over the course of the study period. The 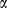
′s and 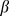 ‘s in the equations represent the coefficients that are to be estimated via regression analysis. Models were estimated with probability weighted data and using clustered standard errors, where clusters were village-years [34]. Clustering by village-year allowed for a large number of clusters (greater than 50), which is required for the use of statistical methods based on asymptotic theory [35].

The specification of these models identify the effect of the copra price increase on copra and fishing labor using variation in the copra price across time and islands (Table 1). We also use household land under coconut cultivation to estimate differences in how the copra price affected households with different levels of capital assets. We chose to use household land because households with more (or less) l and should experience larger (or smaller) increases in income as a result of the copra price increase and because we had greater confidence in our measurement of household land as compared to other forms of household capital. This identification strategy helps address concerns about measurement error and the effect of unobserved aggregate trends. If we observe plausible heterogeneous responses, our concerns that recall bias or aggregate trends in unobserved variables have driven our results will be mitigated. In addition, it provides important information to improve program efficiency by helping to identify which households respond most strongly to the copra price change.

To show how the copra price change affected copra labor and fishing labor across households with difference areas of land in coconut production, we used the coefficient estimates from the regression analysis to plot the following equations for the elasticity of copra labor and fishing labor with respect to the copra price (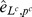,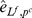, respectively):
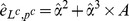


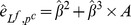



To estimate the population-level effect of the copra price change on copra labor and fishing labor, we estimated the sample mean elasticity of fishing labor and copra labor with respect to the copra price, given the distribution of household land in the sample of households at the start of the study (2001). Specifically, we calculated 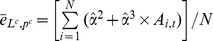 and 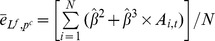. We used these expressions to calculate the estimated percentage change in labor based on the known percentage change in the copra price.

We also checked the robustness of the regression analysis results by estimating two sets of additional model specifications. First, we estimated models that were identical to the main models with two different variations: 1) additional control variables and 2) only using household land from the first year of the study in the interaction term with the copra price. Second, we estimated models 1) without the interaction between copra price and household land and 2) without the interaction between copra price and household land, and with additional control variables.

In addition, to corroborate the trends in labor that we estimated, we estimated the effect of the copra price on copra and fishing income as well as on spending on fish and rice using the main model specifications used for estimating labor.

### Ecological Data and Analysis

In order to predict the effect of estimated changes in fishing labor on ecological stocks, we conducted detailed fishing surveys with 145 additional households and underwater visual surveys at 37 reef sites across an extreme spatial gradient in fishing labor on one island, Kiritimati (Fig. 1) [32].

Fishing effort on Kiritimati was estimated as the density of fishing effort along a stretch of coastline and ranged from 947 hrs/km/wk at some reef sites near the largest villages to no reported fishing on reefs along the unpopulated coast (Table 3). This estimate of fishing effort corresponds to approximately 50 households fishing one kilometer of coastline for about 20 hours per week in the most heavily fished areas. The total range of biomass of fish observed across this fishing gradient was from over 8 mT/ha to less than a quarter of a metric ton per hectare, which is approximately the range observed between the few pristine coral reefs in the Pacific to the heavily degraded coral reefs in the Caribbean [38] (Table 3). Similarly, the reefs ranged from being almost totally dominated by reef building organisms (corals and crustose coralline algae) (91% of the coral reef area) to reefs that have been almost entirely overgrown by algae (macroalgae and turf algae) (80% of the coral reef area) (Table 3).

**Table 3:** Descriptive statistics of fishing and ecological survey data from Kiritimati (2007).

Variable	[units]	Mean	N	SD	Max	Min
Std. Fishing Effort	[hrs/km/wk]	275	37	294	947	0
Total Fish	[mT/ha]	2.43	37	2.07	8.28	0.17
Herbivorous Fish	[mT/ha]	0.51	37	0.22	1.11	0.00
Algae	[cover]	0.35	37	0.19	0.80	0.08
Reef	[cover]	0.61	37	0.19	0.91	0.17

We examined the potential impacts of changes in fishing labor (standardized for gear type) on key coral reef ecological groups using path analysis. In these models, fishing may directly affect fish stocks and indirectly affect corals, and hence coral reef ecosystem services, through reductions in herbivorous fish stocks or stocks of all fish species. Herbivorous fish help maintain the balance between corals and their algal competitors through grazing [27], [29], [39]. In addition, fishing of any species may increase the nutrients available to algae because fish are important sinks for nutrients [40]. These pathways are represented by the following set of equations: 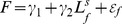


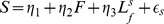


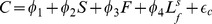
where 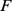
is the total abundance of fish, 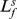 is the fishing effort at the reef site, 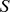 is algae, and 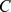 are reef-building organisms. The 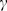
′s, 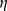
′s, and 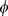
′s in the equations represent the coefficients that are to be estimated via regression analysis. These models were estimated using ordinary least squares and parameter estimates from these models were used to estimate the effect of the change in fishing labor due to the copra price increase on reef-builders.

We argue that this is a valid approach because the location of fishing effort is determined exogenously by the government through historical village planning and fishing effort is restricted to reefs near villages because few households own canoes or automobiles [18], [32]. Importantly, we have no evidence that the government's village planning decisions were based on fine scale differences in fishing productivity that could affect the marginal productivity of fishing labor. However, if the government did settle villages in areas of high fisheries productivity, our results would underestimate the effect of fishing on fish stocks because the historic biomass of fish on heavily fished reefs would have been even higher than on the lightly fished reefs we observe. In addition, although corals and algae may have some effect on fish abundance, empirical evidence shows that top-down effects of fish on corals and algae dominate [18], [28].

## Results

### Labor Allocation

**Figure 2:**
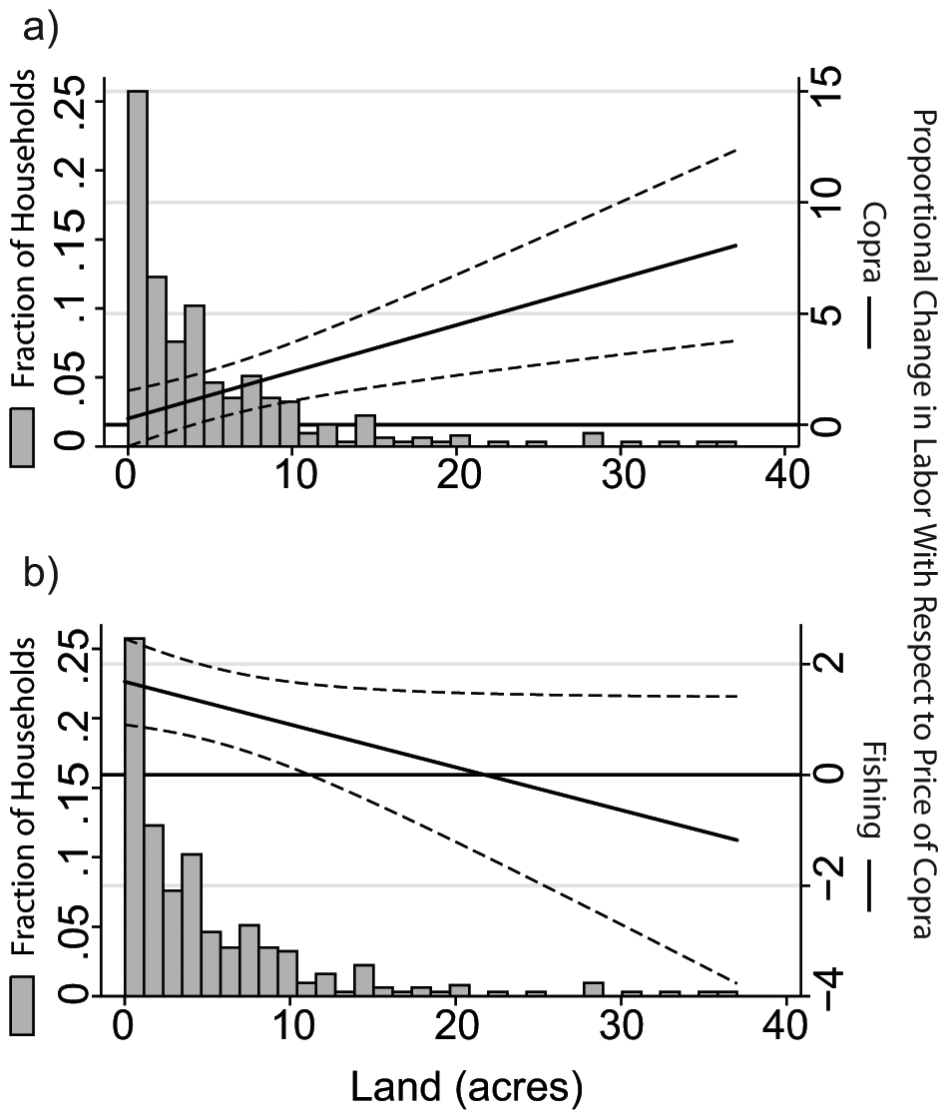
Effect of the copra price increase on labor. Empirical estimates of the elasticity of copra labor (a) and fishing labor (b) with respect to the copra price for different levels of household land under coconut cultivation.

The results of our regression analysis suggest that the copra price resulted in an increase in copra labor but this effect was significant only for households with larger areas of land under coconut cultivation (Table 4, Fig. 2). This is indicated by the insignificant main effect of the copra price (0.277, *SE* = 0.639, p>0.1), the positive significant effect of the copra price interacted with household land under coconut cultivation (0.210, *SE* = 0.065, p<0.01), and the significance of the sum of these effects at larger levels of land (Fig. 2). Using these coefficient estimates together to calculate the price elasticity of copra labor with respect to the copra price, given the distribution of household land, we estimate a mean price elasticity of 1.076 (*SE* = 0.081, p<0.0001). This suggests that the 29.7% copra price increase may have increased copra labor by 31.7%.

**Table 4:** Estimates of fishing labor 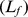 and copra labor 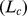 ^1^.

Variable	ln(*L_f_*)	ln(*L_c_*)
ln(*p_c_*)	1.683***	0.277
	[0.397]	[0.639]
ln(*p_f_*)	0.034	0.057
	[0.778]	[0.843]
Land (*A*)	−0.071	0.009
	[0.051]	[0.116]
ln(*p_c_*)x*A*	−0.077*	0.210***
	[0.040]	[0.065]
HH Size	0.118***	−0.061
	[0.040]	[0.076]
Males	0.389***	0.045
	[0.114]	[0.176]
Education	−0.135	0.284
	[0.086]	[0.184]
Rain_(*t*−1+*t*−2)_	−0.000*	0
	[0.000]	[0.000]
Constant	0.614	5.767
	[1.678]	[4.021]
Observations	1574	1627
Island FE	YES	YES
HH FE	YES	YES

1 Robust standard errors in brackets. *p<0.1, **p<0.05, ***p<0.01.

The regression analysis also suggests that the copra price increase resulted in an increase in fishing labor and that this was smaller for households with larger areas of land under coconut cultivation (Table 4, Fig. 2). This is indicated by a positive main effect of the copra price (1.683, *SE* = 0.397, p<0.01) and a negative effect of the copra price interacted with household land under coconut cultivation (−0.077, *SE* = 0.040, p<0.1). The mean price elasticity of fishing labor with respect to copra price, based on these coefficient estimates and household land, is 1.288 (*SE* = 0.030, p<0.0001). This suggests that the copra price increase resulted in a similar level of increase in fishing labor (38.2%) as compared to the increase in copra labor.

The signs on the control variables in the fishing labor model are generally consistent with predictions from economic theory, which gives us greater confidence in these results. For example, larger households with more men had higher levels of fishing labor. However, fish price is not a significant predictor of fishing labor and no control variables in the copra labor model were found to be significant, which could be a result of measurement error. It should be noted, also, that 63% and 56% of households reported no change in copra labor and fishing labor, respectively, over this time period, which means that these patterns in labor are being identified off of less than 200 households.

Our estimates from additional model specifications provide another set of evidence to evaluate our results (Table S2, S3). First, the results for both the fishing and copra labor models were robust to the inclusion of additional variables that may affect the shadow wage rate, including reef area, house type, rain in the current year, and fishing capital (Table S2, S3). Current rainfall was used to control for changes in the available number of days to dry coconut meat (one step in the copra production process) and bad weather days that may decrease the likelihood of going fishing. Second, these results are most likely not being affected by endogenous land area investment decisions because the model specification using land in the first year in place of current year land resulted in almost identical estimates (Table S2, S3). Third, models with the main effects of copra price only and no interaction with land holdings showed similar results to the models with main and interactive effects. In the case of fishing labor (Table S2), the copra price also had a positive but smaller effect, which is consistent with the negative sign on the interaction term in our primary model. Controls such as household size and number of working aged males also had coefficients of similar magnitude and sign. These results were also robust to the inclusion of additional control variables. In the case of copra labor (Table S3), the copra price was still insignificant; however, the effect of the price of fish on copra labor was positive and significant, which could suggest some effect of copra labor on demand for fish.

Our estimates of the effect of the copra price increase on copra and fishing income as well as spending on fish and rice corroborate the results for our estimates of labor (Table S4, Table 5). Income from copra increases with the largest increases for households with large amounts of land, while income from fishing did not change at all or decreased slightly for households with the largest amount of land (note that fishing labor may increase without the sale of fish increasing) (Table 5). Spending on both fish (including local and imported canned fish, which can be considered a luxury) and rice increases, with spending on rice increasing the most (Table 5).

**Table 5:** Elasticity of labor, income, and spending with respect to copra price^1^.

Variable		Elasticity	SE	95% Conf. Interval	
Labor					
	Copra	1.076***	0.081	0.917	1.236
	Fishing	1.288***	0.030	1.230	1.347
Income					
	Copra	1.668***	0.086	1.5	1.83
	Fishing	−0.302***	0.056	−0.41	−0.19
Spending					
	Fish	2.348***	0.008	2.33	2.36
	Rice	4.825***	0.323	4.19	5.46

1 Elasticities are calculated based on coefficient estimates from Table 4 (for labor), Table S4 (for income and spending), and the distribution of household land under coconut cultivation. Robust standard errors. *p<0.1, **p<0.05, ***p<0.01.

Note that we also estimate copra production using the same specifications as in the main model for copra labor; however, we found no effect of copra price on copra production. We also tested alternative model specifications, including models with one and two year lags in the copra price and instrumental variables regression with copra labor instrumented by the price of copra, land, and their interaction. We found no effects of copra price or the instrumented copra labor with these alternative specifications. We attribute this to substantial measurement error in copra production. This seems like a reasonable explanation given that households often reported production by number of bags (which was then used to estimate kilograms) rather than kilograms.

### Ecosystem Effects

**Figure 3:**
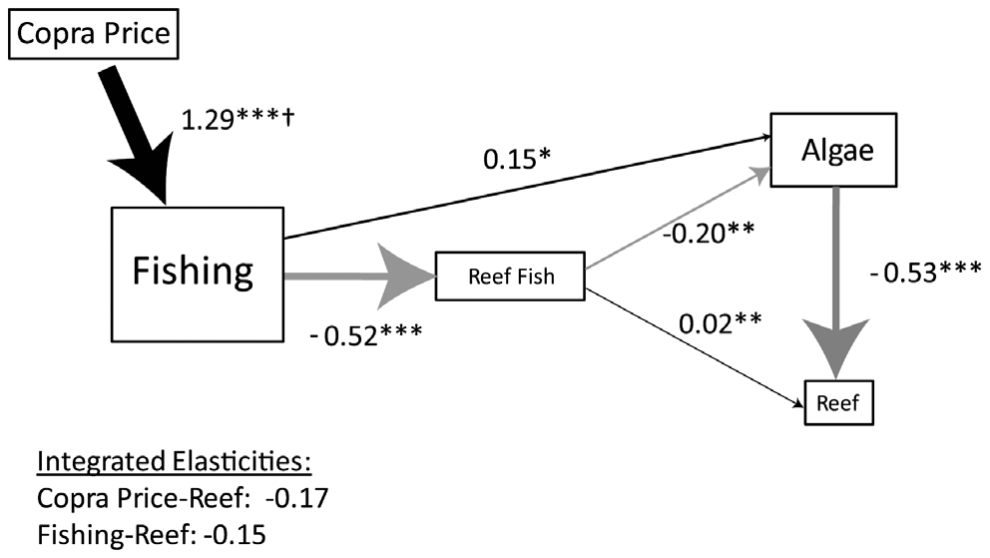
Effect of the increase in the copra price and fishing on the coral reef ecosystem. Ecosystem models and estimates of elasticities from a path analysis of the effect of fishing on the coral reef ecosystem, occurring primarily through changes in (a) total fish biomass and (b) biomass of herbivorous fish only. * p<0.1, **p<0.05, ***p<0.01+ Based on econometric results. The relative size and color (black: positive, gray: negative) represent the magnitude and sign of the effect.

The results of the ecological path analysis suggest that changes in fishing that resulted from an increase in the copra price may have had indirect negative effects on coral reef-building organisms (Fig. 3). Unsurprisingly, we find that fishing at the reef sites negatively affects the total biomass of fish at the reef sites (Fig. 3). Total fish biomass has direct negative effects on algae (Fig. 3) and small positive direct effects on reef-builders at the reef sites (Fig. 3). Algae had negative effects on reef-builders (Fig. 3). Combining the results from the path analysis and the model of fishing labor, we predict that the 29.7% increase in the copra price may have resulted in a 19.9% decrease in total fish stocks and indirectly resulted in a 4.5% decrease in reef-builders due to changing ecological interactions.

## Discussion

Our results suggest that the copra price increase in Kiribati not only directly affected copra labor but that it also affected fishing labor and the health of the coral reef ecosystem. Moreover, the impacts of the price increase varied across households with different levels of land as well as across different parts of the coral reef ecosystem, such as fish, algae, and corals. Although these results represent just one case study involving a relatively small number of households, they suggest that economic development policies and programs may have far-reaching ecological and economic consequences that often go overlooked or at least unmeasured [10].

Our estimate of an increase in copra labor is consistent with standard economic theory as well as a sustainable livelihood framework [5]. Although we appear to have noisy estimates for the main effect of copra price on copra labor, we estimate significant positive effects of the copra price on copra labor when we are able to identify households where the copra price increase is likely to make the most impact (i.e., households with more land). These results suggest that households with larger areas of land may have increased copra labor in response to the copra price because the copra price increase resulted in a larger increase in the marginal revenue product of labor in copra. In contrast, households with a small amount of land may not have increased copra labor because they were constrained by land. Measurement error and insufficient data may also explain the lack of a significant main effect of the copra price on copra labor. Importantly, though, these results for changes in copra labor were corroborated by evidence of similar trends in copra income.

Our estimate of an increase in fishing labor as a result of the copra price increase is not consistent with predictions from a standard economic model, and supports the proposition that economic development policies may sometimes have unintended and unexpected impacts beyond the sector they were design to target [7], [24]. Our estimate of an increase in fishing labor for households with small areas of land under coconut cultivation is not consistent with simple income effects, suggesting that other mechanisms may be driving these empirical results. Interestingly, this increase in fishing labor for households with a small amount of land did not result in an increase in fishing income, which suggests that fishing was for consumption and/or non-monetary benefits. In contrast, households with large amounts of land that increased copra labor and income had a concomitant decrease in fishing income, although we did not find a significant decrease in fishing labor for these households, which could be explained by the small number of households with large areas of land under coconut cultivation.

Previous studies and economic theory suggest at least four potential mechanisms by which the copra price increase may have resulted in an increase in fishing labor: 1) additional income increases the demand for local resources and draws labor back into resource extractive activities through general equilibrium effects (e.g. effects that occur through interactions of multiple interacting markets), 2) additional income is reinvested in fishing capital [20], increasing the marginal productivity of labor in the resource extractive activity, 3) fish stocks are declining, causing fishermen to increase effort and this trend is correlated with the copra price increase, and 4) additional income increases fishing because fishing has important non-monetary benefits [15], [41]. Our observations find little to no support the first three mechanisms, while, in contrast, we found various lines of evidence that suggest that fishing has important non-monetary benefits in Kiribati. Contrary to the conditions of this first mechanism, we observed a decrease in fishing income. However, we do observe a small increase in the fish price and large increase in spending on fish. Importantly, spending on canned fish could explain the limited increase in fish price and the non-positive trend in fish income and suggests that increased demand may have more of a limited effect on local fishing labor. We also did not observe any significant changes in fishing capital due to the copra price increase that would provide evidence for the second mechanism. Although there is some evidence that the fish stocks in some parts of Kiribati are declining, overall they are relatively healthy and we have no reason to believe that the pattern of decline across years and islands corresponds to the changes in the copra price. In support of the third mechanism, we found that when households were asked if they were satisfied with their income or if they wanted to find other sources of income, 38% (95% CI: 28%, 38%) of the households that were not satisfied with their income indicated that they wanted to improve fishing. Importantly, for these households, fishing is probably not the occupation of last resort [13], [42]. Instead, qualitative evidence suggests that fishing is a culturally important and personally desirable occupation in Kiribati [43]; and, as a result, fishing is more widely respected than the few other more highly paid livelihoods.

The importance of non-monetary benefits from livelihoods is well-recognized by anthropologists and some economists (e.g., [14], [15], [44], [45], [46]). These effects have not been formally examined in models of fishing or other rural households. However, in a model of labor supply in a developed country context, Farzin [41] shows that non-monetary benefits may substitute for wages under certain conditions and result in a labor supply curve that is not predicted by standard economic theory. We verified that non-monetary benefits from fishing may also, in theory, result in a non-standard labor supply curve under conditions that exist in Kiribati by exploring a simple theoretical model of a fishing-agricultural household (See Text S1). With this model, it is possible to show that there is an increase in fishing as a result of a copra price increase if consumer goods and leisure do not easily substitute for non-monetary benefits from fishing. However, future research is needed to develop and empirically test a detailed model of the role of non-monetary benefits in fishing-agricultural household livelihood decisions.

Our relatively small sample size and use of re-call data suggest caution in drawing strong conclusions from our analysis of labor decisions. These limitations of the data could explain the two largest concerns from the empirical analysis of changes in labor: 1) the lack of effect of fish price on fishing labor and 2) the large number of households that reported no change in labor. Fish price is reported in different units across species, households, and islands, which we had to convert to a common unit of measure. This may have introduced additional measurement error on top of the measurement error coming from the small sample. Although the high percentage of households reporting no change in labor suggests recall bias, the fact that households that reported no change in copra labor had smaller land holdings (4.20 acres, *SE* = 0.15) than those that reported changes in copra labor (7.14 acres, *SE* = 0.35) (Mann-Whitney Test, z = 8.34, *p*<0.0001) is consistent with the expectation that households with small land holdings would experience the smallest changes in copra labor. This is because small changes or less intense experiences are more likely to be forgotten [47].

Despite these limitations, the various lines of support for our finding of an increase in fishing labor give us further confidence in our estimation of the ecological consequences of the copra price increase. If in fact fishing did increase, the copra price increase may have unexpected long-term negative consequences for the welfare of the household that outweigh the benefits of the increase in income. Our projection of a decline in fish stocks and reef-builders suggests long-run losses in fish catch and other coral reef ecosystem services, such as protection from storms [48]. Importantly, our estimate of losses in reef-builders is probably conservative because reef-builders are slower to respond to changes than fish or algae [25]. On historically over-fished reefs, sudden and almost complete losses of reef-builders have been observed following disturbances, such as hurricanes [49].

## Conclusions

By empirically examining the direct and indirect consequences of an agricultural price increase in the Republic of Kiribati, we uncovered potentially negative effects on the fishery and the coral reef ecosystem. Neither the Ministry of Finance that implemented the copra price increase nor the Ministry of Fisheries who supported the policy expected these negative consequences. These findings highlight the importance of taking a systems approach and working across government agencies in order to better design policies to meet economic and environmental objectives [3].

## Supporting Information

Text S1 Household model with non-monetary benefits from fishing. Using a model of labor supply in a developed country context, Farzin [41] shows that non-monetary benefits may substitute for wages under certain conditions and result in a labor supply curve that is not predicted by standard economic theory. We verify that non-monetary benefits from fishing may also, in theory, result in a non-standard labor supply curve under conditions that exist in Kiribati. Using a simple theoretical model of a fishing-agricultural household, it is possible to show that there is an increase in fishing as a result of a copra price increase if consumer goods and leisure do not easily substitute for non-monetary benefits from fishing.
(DOC)

Table S1 Spearman rank correlations between prices (*p*), labor (*L*), income (*I*), and other household (HH) survey data.
(DOCX)

Table S2Estimates of fishing labor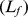 from additional model specifications.(DOCX)

Table S3Estimates of copra labor 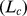 from additional model specifications.(DOCX)

Table S4Estimates of copra income (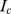), fishing income (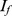), spending on fish (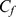), and spending on rice (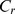).(DOCX)

## References

[1] UN Millenium Project (2005) Investing in Development: A Practical Plan to Achieve the Millenium Development Goals. Washington, D.C.

[2] MEA (2005) Millennium ecosystem assessment synthesis report: Millennium Ecosystem Assessment.

[3] NRC (2013) Sustainability for the Nation: Resource Connection and Governance Linkages: The National Academies Press.

[4] Alberti M, Asbjornsen H, Baker LA, Brozovic N, Drinkwater LE, et al (2011) Research on Coupled Human and Natural Systems (CHANS): Approach, Challenges, and Strategies. Bulletin of the Ecological Society of America 92: 218–228.

[5] Allison EH, Ellis F (2001) The livelihoods approach and management of small-scale fisheries. Marine Policy 25: 377–388.

[6] Ellis F (1998) Household strategies and rural livelihood diversification. The Journal of Development Studies 35: 1–38.

[7] Allison EH, Horemans B (2006) Putting the principles of the sustainable livelihoods approach into fisheries development policy and practice. Marine Policy 30: 757–766.

[8] Dasgupta P (2001) Human Well-Being and the Natural Environment. Oxford, UK: Oxford University Press.

[9] Scheffer M, Carpenter S, de Young B (2005) Cascading effects of overfishing marine systems. Trends in Ecology & Evolution 20: 579–581. 16701438 10.1016/j.tree.2005.08.018

[10] Kremen C, Merenlender A, Murphy D (1994) Ecological monitoring: a vital need for integrated conservation and development projects in the tropics. Conservation Biology 8: 388–397.

[11] Angelsen A (1999) Agricultural expansion and deforestation: modelling the impact of population, market forces and property rights. Journal of Development Economics 58: 185–218.

[12] Bluffstone RA (1995) The effect of labor market performance on deforestation in developing countries under open access: an example from rural Nepal. Journal of Environmental Economics and Management 29: 42–63.

[13] Pendleton L, Howe E (2002) Market integration, development, and smallholder forest clearance. Land Economics 78: 1–18.

[14] Gatewood J, McKay B (1990) Comparison of job satisfaction in six New Jersey fisheries: implications for management. Human Organization 49: 14–25.

[15] Pollnac R, Bavinck M, Monnereau I (2012) Job Satisfaction in Fisheries Compared. Social Indicators Research 109: 119–133. 22997480 10.1007/s11205-012-0059-z PMC3439617

[16] Swift J (1989) Why are rural people vulnerable to famine? IDS bulletin 20: 8–15.

[17] Christensen NL, Bartuska A, Brown JH, Carpenter S, D'Antonio C, et al (1996) The report of the Ecological Society of America Committee on the scientific basis for ecosystem management. Ecological Applications 6: 665–691.

[18] Sandin S, Smith J, DeMartini E, Dinsdale E, Donner S, et al (2008) Degradation of coral reef communities across a gradient of recent human disturbance. PLoS ONE 3: e1548. 18301734 10.1371/journal.pone.0001548 PMC2244711

[19] Mumby PJ, Harborne AR, Williams J, Kappel CV, Brumbaugh DR, et al (2007) Trophic cascade facilitates coral recruitment in a marine reserve. Proceedings of the US National Academy of Sciences 104: 8362–8367. 10.1073/pnas.0702602104 PMC1895955 17488824

[20] Sievanen L, Crawford B, Pollnac R, Lowe C (2005) Weeding through assumptions of livelihood approaches in ICM: seaweed farming in the Philippines and Indonesia. Ocean & Coastal Management 48: 297–313.

[21] Scoones I (1998) Sustainable rural livelihoods: a framework for analysis. Brighton, UK: Institute of Development Studies (IDS) Working Paper 72.

[22] Muller J, Albers H (2004) Enforcement, payments, and development projects near protected areas: how the market setting determines what works where. Resource and Energy Economics 26: 185–204.

[23] Liese C, Smith M, Kramer R (2007) Open access in a spatially delineated artisanal fishery: the case of Minahasa, Indonesia. Environment and Development Econonomics 12: 123–143.

[24] Sauni S, Sauni L, Power M (2005) Fishy tales from Kiribati: declining resources, population growth a worry. Island Business.

[25] Crépin A-S (2007) Using fast and slow processes to manage resources with thresholds. Environmental and Resource Economics 36: 191–213.

[26] Moberg F, Folke C (1999) Ecological goods and services of coral reef ecosystems. Ecological Economics 29: 215–233.

[27] Hughes TP (1994) Catastrophes, phase shifts, and large-scale degradation of a Caribbean coral reef. Science 265. 10.1126/science.265.5178.1547 17801530

[28] Newman M, Paredes G, Sala E, Jackson J (2006) Structure of Caribbean coral reef communities across a large gradient of fish biomass. Ecology Letters 9: 1216–1227. 17040324 10.1111/j.1461-0248.2006.00976.x

[29] Smith J, Hunter C, Smith S (2010) The effects of top-down versus bottom-up control on benthic coral reef community structure. Oecologia 163: 497–507. 20058024 10.1007/s00442-009-1546-z

[30] Carilli J, Walsh S (2012) Benthic foraminiferal assemblages from Kiritimati (Christmas) Island indicate human-mediated nutrification has occurred over the scale of decades. Marine Ecology Progress Series 456: 87–99.

[31] Dinsdale EA, Pantos O, Smriga S, Edwards RA, Angly F, et al (2008) Microbial ecology of four coral atolls in the Northern Line Islands. Plos One 3: e1584. 18301735 10.1371/journal.pone.0001584 PMC2253183

[32] Walsh SM (2011) Ecosystem-scale effects of nutrients and fishing on coral reefs. Journal of Marine Biology.

[33] Huffman G (2001) Global precipitation at one-degree daily resolution from multisatellite observations. J Hydrometeor 2: 36–50.

[34] MaCurdy T (2007) Chapter 62 A Practitioner's Approach to Estimating Intertemporal Relationships Using Longitudinal Data: Lessons from Applications in Wage Dynamics In: Handbook of Econometrics. Heckman J, Leamer E, editors. Amersterdam: North Holland Publishing Co. pp. 4057–4167.

[35] Angrist J, Lavy L (2002) The Effect of High School Matriculation Awards: Evidence from Randomized Trials. No. w9389. National Bureau of Economic Research.

[36] Catala R (1957) Report on the Gilbert Islands: Some Aspects of Human Ecology. Atoll Research Bulletin 59: 1–187.

[37] NRC (1951) Handbook for Atoll Research. Washington, D.C.: National Research Council.

[38] Knowlton N, Jackson J (2008) Shifting baselines, local impacts, and global change on coral reefs. PLoS Biology 6: 215–220. 10.1371/journal.pbio.0060054 PMC2253644 18303956

[39] McCook L, Jompa J, Diaz-Pulido G (2001) Competition between corals and algae on coral reefs: a review of evidence and mechanisms. Coral Reefs 19: 400–417.

[40] Kitchell J, O'Neill R, Webb D, Gallepp G, Bartell S, et al (1979) Consumer regulation of nutrient cycling. BioScience 29: 28–34.

[41] Farzin YH (2009) The effect of non-pecuniary motivations on labor supply. The Quarterly Review of Economics and Finance 49: 1236–1259.

[42] Baland J-M, Francois P (2005) Commons as insurance and the welfare impact of privatization. Public Economics 89: 211–231.

[43] Walsh SM (2009) Linking coral reef health and human welfare [Ph.D.]. La Jolla: University of California - San Diego.

[44] Apostle R, Kasdan L, Hanson A (1985) Work satisfaction and community attachment among fishermen in southwest Nova Scotia. Canadian Journal of Fisheries and Aquatic Sciences 42: 256–267.

[45] Pollnac R, Poggie Jr M (1988) The structure of job satisfaction among New England fishermen and its application to fisheries management policy. American Anthropologist 90: 888–901.

[46] Smith C (1981) Satisfaction bonus from salmon fishing: implications for economic evaluation. Land Economics 57: 181–196.

[47] Lee M (2005) Micro-econometrics for policy, program, and treatment effects: Oxford University Press. 263 p.

[48] Beck MW, Shepard C (2012) Coastal Habitats and Risk Reduction. In: Works AD, editor. World Risk Report. Berlin: Bundnis Entwicklung Hilft.

[49] Knowlton N (1992) Thresholds and multiple stable states in coral reef community dynamics. American Zoologist 32: 674–682.

