# Supplementary material for: Consequences of a Government-Controlled Agricultural Price Increase on Fishing and the Coral Reef Ecosystem in the Republic of Kiribati
Source: PLoS One. 2014 May 12;9(5):e96817. doi: 10.1371/journal.pone.0096817 (PMC4018407; doi:10.1371/journal.pone.0096817)
Supplement: Text S1 — Household model with non-monetary benefits from fishing. Using a model of labor supply in a developed country context, Farzin [41] shows that non-monetary benefits may substitute for wages under certain conditions and result in a labor supply curve that is not predicted by standard economic theory. We verify that non-monetary benefits from fishing may also, in theory, result in a non-standard labor supply curve under conditions that exist in Kiribati. Using a simple theoretical model of a fishing-agricultural household, it is possible to show that there is an increase in fishing as a result of a copra price increase if consumer goods and leisure do not easily substitute for non-monetary benefits from fishing. (DOC) [file pone.0096817.s001.doc]

**Text S1**

**Household Model with Non-Monetary Benefits from Fishing**

We developed a simple theoretical model of a fishing-agricultural household in Kiribati that derives non-monetary benefits from fishing in order to demonstrate whether a copra price increase could result in a fishing price increase under conditions normally experienced in Kiribati. In Kiribati, consumption and labor supply opportunities are severely limited, so we start by assuming limited availability of product and labor markets. We assume that households are endowed with $A$ units of land and $T$ units of time. Households maximize a utility function defined over rice ($c_{r}$), fish ($c_{f}$ ), leisure ($l$), and time spent fishing ($L_{f}$). This specification of the utility function represents our assumption that households in Kiribati derive some non-monetary benefits from fishing. Income is produced through labor in copra $L_{c}$ and time spent fishing $L_{f}$, with the production functions for copra and fishing being $g(A,L_{c})$and $f(L_{f})$, respectively. Copra is by and large exported, so we abstract from domestic consumption of copra. We normalize the price of rice to one, while the price of fish is $p_{f}$and the price of copra is $p_{c}$. With no opportunities for hiring labor from outside the household, or supplying own labor outside of the household, we have $l+L_{f}+L_{c}=T$.

Intuitively, an increase in the government-set copra price ($p_{c}$) has two main effects: first, it increases the marginal revenue product of copra labor at any given level of copra labor; second, it increases household income. The former effect increases the shadow price of leisure and the shadow price of fishing effort. This encourages households to increase labor in copra and reduce leisure and time spent fishing. The latter effect will increase consumption of all normal goods, including time spent fishing. Because of these countervailing effects, the net effects on copra labor and time spent fishing are theoretically ambiguous. For some sets of preferences and production functions, an increase in the copra price can increase time spent fishing. For this effect to emerge, the fact that $L_{f}$ directly enters the utility function (i.e. that households derive non-monetary benefits from fishing) is a necessary, but not sufficient condition.

To illustrate the logic clearly, we can examine specific choices of the utility function and production functions. Suppose the household’s problem from above is given by:

$$\max_{c_{r},c_{f},l,L_{f},L_{c}}\left[\alpha_{1}L_{f}^{\sigma}+(1-\alpha_{1})(c_{f}^{\beta_{1}}c_{r}^{\beta_{2}}l^{1-\beta_{1}-\beta_{2}})^{\sigma}\right]^{1/\sigma}$$

subject to:

$$c_{r}+p_{f}c_{f}=p_{f}f(L_{f})+p_{c}g(A,L_{c})$$

$$l+L_{f}+L_{c}=T$$

$$f(L_{f})=L_{f}^{\gamma}$$

$$g(A,L_{c})=A^{1-\delta}L_{c}^{\delta}$$

and the standard non-negativity constraints on consumption, leisure, and labor supplies.

The parameter $\sigma$ in the constant elasticity of substitution (CES) utility function we employ indexes the degree of substitutability between $L_{f}$ and the other goods. When $\sigma$ is close to 1, the sets of goods are close to perfect substitutes. In this case, when the price of copra increases, households will tend to reduce time spent fishing: the shadow price of fishing increases, and utility gains can come easily from spending the additional income on consumption and leisure. In contrast, low values of $\sigma$ indicate poor substitutes, with especially negative values producing Leontief-like preferences . Leontief preferences describe a situation where a consumer will only have higher utility if the quantities of all goods increase. A classic example is that of consuming shoes; more right shoes do not increase utility without more left shoes. In this case, spending additional income on consumption and leisure, while reducing fishing, is not necessarily optimal because a low level of fishing constrains the gains from increasing consumption and leisure.

We illustrate these ideas by choosing different values of σ, to represent cases where non-monetary benefits from fishing and other consumption and leisure are 1) good substitutes for all households, 2) poor substitutes for all households, and 3) where preferences vary across households. Each case examines the effect of changing the price of copra from a baseline ($p_{c}$= 1) to a higher level ($p_{c}$= 1.5)[[1]](#footnote-2). We find the optimal choices of $L_{f}$and $L_{c}$ numerically for each case, and graph the elasticity[[2]](#footnote-3) of both labor choices with respect to the copra price change at each value of land ownership (see Solution to Household Model below).

Figure S1 (a) shows the case of good substitutes, $\sigma$= 0.1. In this case, we get the sort of outcomes that are expected by standard economic models. An increase in the copra price results in more labor in copra production and less labor in fishing production, across the range of land ownership. The magnitudes of these changes are smaller at larger values of land ownership. Intuitively, larger landowners see a larger income effect from a given price change. Since we almost have Cobb-Douglas preferences, increased income tends to increase both leisure and fishing labor. This income effect makes the fishing labor declines smaller and the copra labor increases smaller for larger landowners.

Figure S1 (b) shows the case of $\sigma$= −2, where the two sets of goods are poor substitutes. As before, an increase in the copra price is associated with an increase in copra labor, with a higher magnitude at low levels of land ownership. But here, across the range of land ownership considered, the impact of a copra price increase on fishing labor is actually positive and increasing with land ownership. As the copra price increases, every household experiences an income effect, with the largest landowners having the largest income effects. Since fishing labor provides utility and other goods and leisure are poor substitutes, households increase fishing labor.

Lastly, we consider the case of heterogeneity in preferences. Heterogeneity in preferences for the non-monetary benefits from fishing is one possible explanation of the heterogeneous responses in household labor we observed across households with different land levels. We have provided evidence suggesting that most households in Kiribati derive some non-monetary benefits from fishing but the degree to which these non-monetary benefits from fishing substitute or complement other consumption or leisure is hard to measure. However, household land is easily observable and may provide some indication of the importance of non-monetary benefits from livelihoods. Although land is certainly not an ideal indicator, it is plausible that households for whom non-monetary benefits from fishing are most important will be least invested in copra and have low levels of land. In contrast, households with high levels of land may derive more benefits from the consumer goods that only cash incomes from copra can buy. Importantly, there is some support for these suppositions from our household survey data and interviews.[[3]](#footnote-4) Yet, future investigations would benefit from improved indicators of household preferences.

To represent the case where preferences vary across households, we allow $\sigma$ to increase in a linear manner from −1.7 to 0.05 as land increases. When household preferences are heterogeneous, we can simulate a pattern of fishing and copra labor that is similar to our empirical results (Fig. S1 c). The impact of the copra price on fishing labor is non-linear, increasing at low levels of land and then decreasing at high levels of land. The impact of the copra price on labor in copra is still positive, but the magnitude is now highest at large levels of land ownership. Intuitively, larger landowners still see the larger income effects, but they also care less for fishing time. The relatively lower “need” for fishing time by large landowners produces a fall in fishing effort and an increase in copra labor.

In sum, this model formalizes one potential explanation for the counter-intuitive effects of the copra price increase in Kiribati.


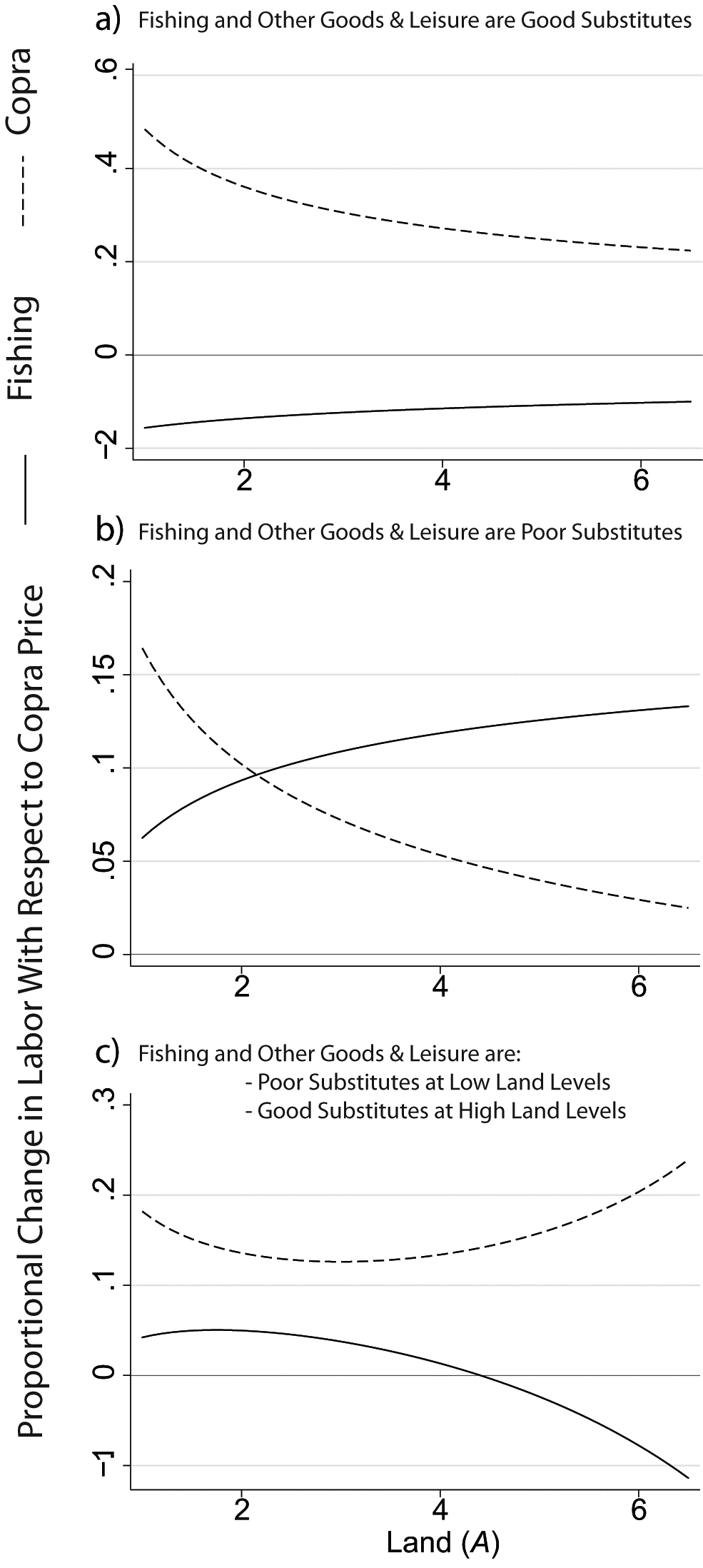


Fig. S1. Simulation results for changes in copra labor and fishing labor with respect to changes in copra price for different levels of land and preferences.

**Solution to Household Model**

Here, we provide details on the solution of the household model. To begin, note that for $\sigma<0$, the value of the utility function approaches zero as any of the choices entering utility approach zero. Since indirect utility is positive at any interior solution for all choices, it is clear that the optimal values of these choices must be non-zero. Therefore, the only corner solution to worry about is where $L_{c}=0$. We first discuss the interior solution and then discuss this corner solution. In both cases, we use a simplification allowed by the parameters given in the text. Given the choices of $\beta_{1}$, $\beta_{2}$, and $p_{f}$ above, $c_{r}$ and $c_{f}$ enter the household maximization problem symmetrically. Therefore, in both the interior solution and the corner solution, $c_{f}=c_{r}=(1/2)\times\left[f(L_{f})+g(A,L_{c})\right]$.

For an interior solution for all choices, these simplifications imply that the problem is equivalent to:

One can set the first-order conditions for the labor choices equal to zero. Manipulating these first-order conditions yields the non-linear system:

These equations can be solved computationally to obtain the interior solution at each parameter value set.

Next, we consider the case where $L_{c}=0$. Note that for an interior solution for consumption, we now have . Also, $l=T-L_{f}$. Therefore, the optimization problem is equivalent to:

After dropping a non-zero multiplicative term, the first-order condition implies:

We can solve this non-linear equation for $L_{f}$ computationally, and then calculate the indirect utility function.

For the parameter values we have chosen, the interior solution always yields a higher optimal value than the corner solution with $L_{c}=0$. Therefore, for the figures we compute elasticities using the interior solutions.

**Reference**

1. Mas-Colell A, Whinston MD, Green JR (1995) Microeconomic theory: Oxford university press New York.

1. For all cases, we use the following values for the parameters: = 7, = 1, = = , = 0.3, = 0.3, and = 0.7. [↑](#footnote-ref-2)
2. To compute this elasticity, we simply calculate the relative change in the labor choice, and divide by 0.5 (for a 50% change in the copra price). [↑](#footnote-ref-3)
3. Households with higher land holdings spend more of their time doing copra (r=.39, p<0.0001) and less time fishing (r=-0.30, p<0.0001). These households have higher cash incomes because, recall, that copra is a cash crop sold for export while fishing is primarily for consumption. [↑](#footnote-ref-4)
