# Supplementary material for: Consequences of a Government-Controlled Agricultural Price Increase on Fishing and the Coral Reef Ecosystem in the Republic of Kiribati
Source: PLoS One. 2014 May 12;9(5):e96817. doi: 10.1371/journal.pone.0096817 (PMC4018407; doi:10.1371/journal.pone.0096817)
Supplement: Table S1 — Spearman rank correlations between prices (p), labor (L), income (I), and other household (HH) survey data. (DOCX) [file pone.0096817.s002.docx]

| Variable | [Units] | $p_{c}$ | $p_{f}$ |
| --- | --- | --- | --- |
| $p_{c}$ | [2001 AUD/kg] | 1.00* |  |
| $p_{f}$ | [2001 AUD/kg] | 0.14* | 1.00* |
| $L_{f}$ | [persons*hrs/yr] | 0.01 | -0.20* |
| $L_{c}$ | [persons*hrs/yr] | 0.03 | 0.02 |
| $L_{other}$ | [persons*hrs/yr] | 0.01 | 0.03 |
| $I_{c}$ | [2001 AUD/yr] | 0.08* | -0.15* |
| $I_{f}$ | [2001 AUD/yr] | -0.02 | -0.11* |
| $I_{other}$ | [2001 AUD/yr] | 0.03 | 0.04 |
| HH that sell fish | [count] | -0.02 | -0.17* |
| Spending on rice | [2001 AUD/yr] | 0.08* | 0.00 |
| Spending on fish | [2001 AUD/yr] | 0.07* | 0.09* |
| Coconut Land | [acres] | 0.03 | -0.07* |
| HH Size | [count] | 0.09* | -0.03 |
| Males | [count (15-60yr)] | 0.05 | -0.11* |
| Education | [yrs >primary] | 0.06* | 0.01 |
| Rain | [mm/yr] | -0.18* | 0.17* |
| Rain*_(t-1+t-2)_* | [mm/yr] | 0.37* | 0.19* |
| Reef Area | [km^2^] | -0.05 | -0.04 |
| House | [1=concrete] | -0.01 | 0.09* |
| Boats | [count] | -0.03 | -0.12* |

Table S1. Spearman rank correlations between prices (*p*), labor (*L*), income (*I*), and other household (HH) survey data. Subscripts indicate copra (*c*), fishing (*f*), or other (*other*) labor or income. Note that these correlations do not control for unobserved heterogeneity across households and islands. *p<0.10.
