# Supplementary material for: Consequences of a Government-Controlled Agricultural Price Increase on Fishing and the Coral Reef Ecosystem in the Republic of Kiribati
Source: PLoS One. 2014 May 12;9(5):e96817. doi: 10.1371/journal.pone.0096817 (PMC4018407; doi:10.1371/journal.pone.0096817)
Supplement: Table S2 — Estimates of fishing labor from additional model specifications. (DOCX) [file pone.0096817.s003.docx]

Table S2. Estimates of fishing labor$(L_{f})$. Robust standard errors in brackets. * p<0.1, **p<0.05, ***p<0.01

| VARIABLES | | $\ln(L_{f})$ | | | | | | | | |  |
| --- | --- | --- | --- | --- | --- | --- | --- | --- | --- | --- | --- |
|  |  | Main Effect | | Main Effect  +Extra Controls | | Main+ Interactive  Effect w/ Initial Land | | | Main+ Interactive  Effect+ Extra Controls | |  |
| $\ln(p_{c})$ | | 1.454*** | | 1.519*** | | 1.682*** | | | 1.559*** | | |
|  | | [0.360] | | [0.322] | | [0.397] | | | [0.338] | | |
| $\ln(p_{f})$ | | -0.486 | | -0.400 | | 0.030 | | | -0.310 | | |
|  | | [0.650] | | [0.642] | | [0.776] | | | [0.725] | | |
| Land ($A$) | | -0.020 | | -0.019 | |  | | | -0.028 | | |
|  | | [0.039] | | [0.037] | |  | | | [0.045] | | |
| $\ln(p_{c})$*$A$ |  | |  | |  | | -0.013 | | | |  |
|  | |  | |  | |  | | | [0.034] | | |
| $\ln(p_{c})$* $A_{t=0}$ |  | |  | | -0.077* | | |  |  |  |  |
|  | |  | |  | | [0.040] | | | |  |  |
| HH Size | | 0.118*** | | 0.115*** | | 0.119*** | | | 0.115*** | | |
|  | | [0.040] | | [0.036] | | [0.040] | | | [0.036] | | |
| Males | | 0.389*** | | 0.364*** | | 0.389*** | | | 0.364*** | | |
|  | | [0.115] | | [0.111] | | [0.114] | | | [0.111] | | |
| Education | | -0.128 | | -0.127 | | -0.136 | | | -0.128 | | |
|  | | [0.085] | | [0.080] | | [0.085] | | | [0.080] | | |
| Rain*_(t-1+t-2)_* | | -0.000* | | -0.000* | | -0.000* | | | -0.000* | | |
|  | | [0.000] | | [0.000] | | [0.000] | | | [0.000] | | |
| Rain | |  | | 0.000 | |  | | | 0.000 | | |
|  | |  | | [0.000] | |  | | | [0.000] | | |
| Reef Area | |  | | -0.019*** | |  | | | 0.006*** | | |
|  | |  | | [0.004] | |  | | | [0.002] | | |
| Concrete House | |  | | -0.814 | |  | | | -0.813 | | |
|  | |  | | [0.586] | |  | | | [0.586] | | |
| Boat | |  | | 1.026*** | |  | | | 1.022*** | | |
|  | |  | | [0.212] | |  | | | [0.214] | | |
| Constant | | 0.370 | | 6.850*** | | -2.018** | | | -1.866*** | | |
|  | | [1.644] | | [1.528] | | [0.938] | | | [0.677] | | |
| Observations | | 1,574 | | 1,542 | | 1,574 | | | 1,542 | | |
| Island FE | | YES | | NO | | YES | | | NO | | |
| HH FE | | YES | | YES | | YES | | | YES | | |
