# Supplementary material for: Consequences of a Government-Controlled Agricultural Price Increase on Fishing and the Coral Reef Ecosystem in the Republic of Kiribati
Source: PLoS One. 2014 May 12;9(5):e96817. doi: 10.1371/journal.pone.0096817 (PMC4018407; doi:10.1371/journal.pone.0096817)
Supplement: Table S3 — Estimates of copra labor from additional model specifications. (DOCX) [file pone.0096817.s004.docx]

Table S3. Estimates of copra labor $(L_{c})$. Robust standard errors in brackets. * p<0.1, **p<0.05, ***p<0.01

| VARIABLES |  |  | $\ln(L_{c})$ |  | |  |
| --- | --- | --- | --- | --- | --- | --- |
|  | Main Effect | Main Effect  +Extra Controls | Main+ Interactive  Effect w/ Initial Land | | Main+ Interactive  Effect+ Extra Controls | |
| $\ln(p_{c})$ | 0.914 | 0.933 | 0.272 | | 0.148 | |
|  | [0.610] | [0.699] | [0.638] | | [0.731] | |
| $\ln(p_{f})$ | 1.518* | 1.348 | 0.041 | | -0.419 | |
|  | [0.801] | [0.874] | [0.841] | | [0.869] | |
| Land ($A$) | -0.133 | -0.129 |  | | 0.041 | |
|  | [0.107] | [0.105] |  | | [0.116] | |
| $\ln(p_{c})$*$A$ |  |  |  | | 0.251*** | |
|  |  |  |  | | [0.069] | |
| $\ln(p_{c})$* $A_{t=0}$ |  |  | 0.211*** | |  | |
|  |  |  | [0.065] | |  | |
| HH Size | -0.063 | -0.057 | -0.060 | | -0.054 | |
|  | [0.075] | [0.076] | [0.076] | | [0.076] | |
| Males | 0.053 | 0.050 | 0.046 | | 0.037 | |
|  | [0.176] | [0.176] | [0.176] | | [0.176] | |
| Education | 0.267 | 0.263 | 0.280 | | 0.283 | |
|  | [0.184] | [0.182] | [0.182] | | [0.183] | |
| Rain*_(t-1+t-2)_* | 0.000 | 0.000 | -0.000 | | -0.000 | |
|  | [0.000] | [0.000] | [0.000] | | [0.000] | |
| Rain |  | -0.000 |  | | -0.000 | |
|  |  | [0.000] |  | | [0.000] | |
| Reef Area |  | -0.004 |  | | -0.040*** | |
|  |  | [0.007] |  | | [0.003] | |
| Concrete House |  | -1.705 |  | | -1.744 | |
|  |  | [1.283] |  | | [1.282] | |
| Boat |  | 0.588** |  | | 0.656** | |
|  |  | [0.258] |  | | [0.259] | |
| Constant | 6.548 | -10.424*** | -1.152 | | 1.296 | |
|  | [4.040] | [3.098] | [1.552] | | [1.199] | |
| Observations | 1,627 | 1,595 | 1,627 | | 1,595 | |
| Island FE | YES | NO | YES | | NO | |
| HH FE | YES | YES | YES | | YES | |
