# Supplementary material for: Consequences of a Government-Controlled Agricultural Price Increase on Fishing and the Coral Reef Ecosystem in the Republic of Kiribati
Source: PLoS One. 2014 May 12;9(5):e96817. doi: 10.1371/journal.pone.0096817 (PMC4018407; doi:10.1371/journal.pone.0096817)
Supplement: Table S4 — Estimates of copra income (), fishing income (), spending on fish (), and spending on rice (). (DOCX) [file pone.0096817.s005.docx]

| VARIABLES | $\ln(\mathrm{I}_{c})$ | $\ln(\mathrm{I}_{f})$ | $\ln(\mathrm{C}_{f})$ | $\ln(\mathrm{C}_{r})$ |
| --- | --- | --- | --- | --- |
|  |  |  |  |  |
| $\ln(p_{c})$ | 0.525 | 0.436 | 2.235*** | 0.535*** |
|  | [0.547] | [0.539] | [0.432] | [0.149] |
| $\ln(p_{f})$ | 1.291* | 1.715 | 0.148 | 0.837* |
|  | [0.740] | [1.098] | [0.907] | [0.425] |
| Land ($A$) | -0.016 | -0.045 | -0.010 | -0.049*** |
|  | [0.100] | [0.078] | [0.048] | [0.019] |
| $\ln(p_{c})$*$A$ | 0.223*** | -0.144** | 0.022 | -0.036* |
|  | [0.068] | [0.062] | [0.059] | [0.019] |
| HH Size | -0.072 | -0.014 | -0.096** | 0.047*** |
|  | [0.066] | [0.071] | [0.048] | [0.014] |
| Males | 0.071 | 0.258 | 0.084 | 0.042 |
|  | [0.154] | [0.244] | [0.124] | [0.038] |
| Education | 0.272* | -0.029 | -0.096 | -0.023 |
|  | [0.147] | [0.156] | [0.075] | [0.019] |
| Rain*_(t-1+t-2)_* | 0.000 | 0.000 | -0.000* | 0.000 |
|  | [0.000] | [0.000] | [0.000] | [0.000] |
| Constant | 15.069*** | 7.381*** | 9.799*** | 6.605*** |
|  | [3.441] | [0.940] | [0.671] | [0.279] |
| Observations | 1,620 | 1,498 | 1,584 | 1,574 |
| Island FE | YES | YES | YES | YES |
| HH FE | YES | YES | YES | YES |

Table S4. Estimates of copra income ($\mathrm{I}_{c}$), fishing income ($\mathrm{I}_{f}$), spending on fish ($\mathrm{C}_{f}$), and spending on rice ($\mathrm{C}_{r}$).
